# Supplementary material for: Fermi surface tomography
Source: Nat Commun. 2022 Jul 15;13:4132. doi: 10.1038/s41467-022-31841-z (PMC9287296; doi:10.1038/s41467-022-31841-z)
Supplement: Supplementary file 1 — Supplementary Information [file 41467_2022_31841_MOESM1_ESM.pdf]

Fermi surface tomography

Supplementary Information

ARPES spectrometers record the photoelectron intensity in an angular scale, independent of the spatial orientation of the surface normals. If this orientation does not coincide with the axis of the lens, the resulting contours of the Fermi surface will appear distorted. If the symmetry of the obtained pattern allows to identify the Γ-point, the map can be redrawn in momentum coordinates by standard formulas using rotation matrices. An example of such a recalculation is shown in Fig. S1.

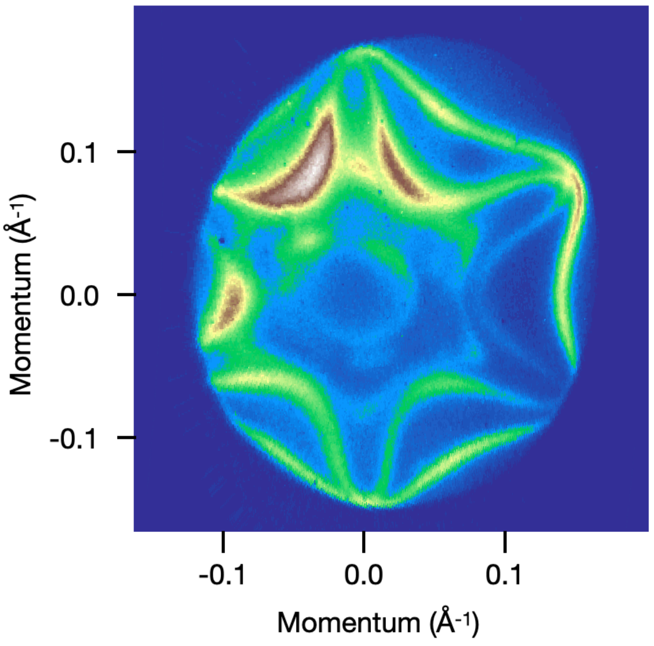

Fig. S1. **Corrected Fermi Surface.** Fermi surface of Bi_2Te_3 from Fig. 3d recalculated in momentum coordinates.

Differentiation of FeSuMa spectra is based on simple subtraction of the intensities recorded at two threshold energies. This operation allows to obtain a number of electrons having kinetic energies between these two values of the threshold energies. In Fig. S2 the example of such subtraction is shown for the threshold energies which are different by 10 meV. Note, that while the two maps on the left look similar due to their integrated intensities reflecting all features in the spectrum within the larger energy interval, their difference bears individual features, such as narrower „petals“ and more homogeneous distribution of maximal intensity.

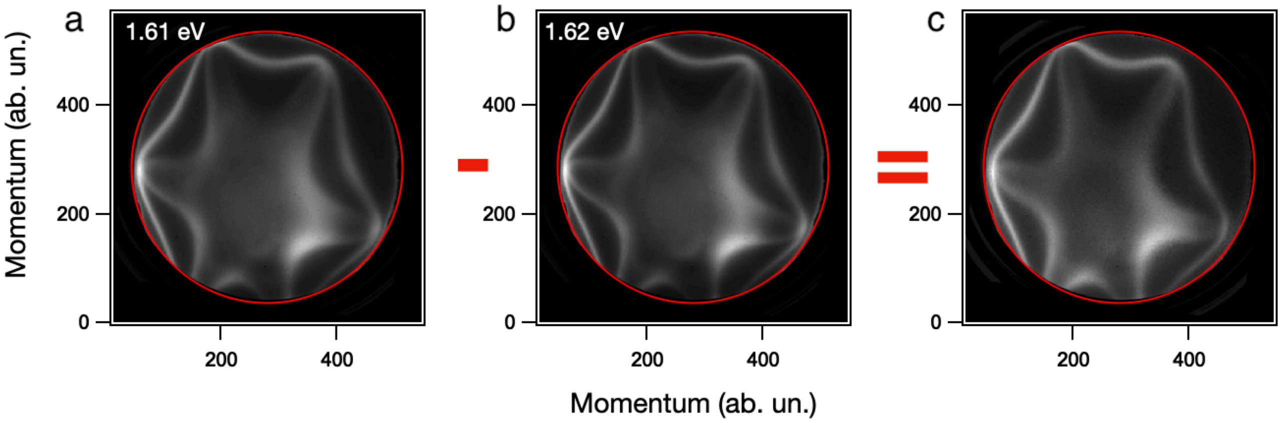

Fig. S2. **Angular distribution at particular energy.** Subtraction of two angular distributions corresponding to two values of threshold energy.

a) Angular distribution corresponding to electrons having kinetic energies more than 1.61 eV.

b) Angular distribution corresponding to electrons having kinetic energies more than 1.62 eV.

c) Angular distribution corresponding to electrons having kinetic energies between 1.61 and 1.62 eV.

Scanning of the threshold energy results in a number of layers which can be collected together into a three-dimensional dataset (see example in Fig. 4). Now one can consider the vertical cuts through this distribution and differentiate the result. We show in Fig. S3 an example of the differentiation of the integrated intensity from such a vertical cut. The result shows conventional dataset, as usually obtained using the hemispherical analyzers.

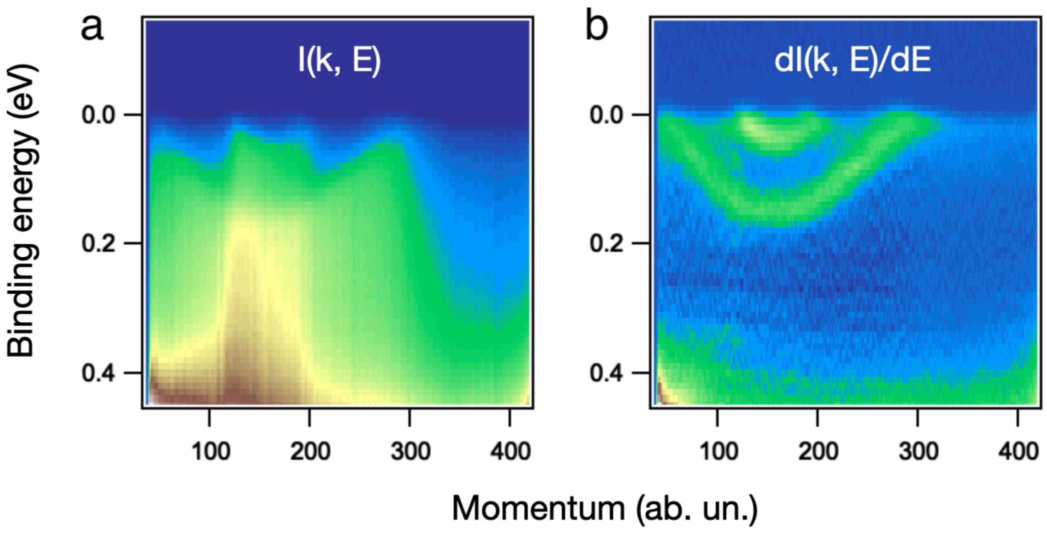

Fig. S3. **Differentiation.** Differentiation of the energy-angle cut from 3D-distribution of integrated intensity.

a) Dataset, as collected by FeSuMa.
b) The same dataset after differentiation.

Although the presently achieved energy resolution is more moderate compared to the best hemispherical analyzers, the angular characteristics of FeSuMa are more advantageous. The angular resolution is anisotropic - there is no entrance slit which cuts only a portion of angular signal and inevitably integrates the intensity in perpendicular direction. The need of signal integration over the slit of finite width can also cause fundamental problems with interpretation of ARPES spectra. In particular, in the case of steeply dispersing Dirac cones in topological insulators and, especially, in graphene, such integration, accompanied by strongly inhomogeneous effects of intensity distribution within the conical band, can even cause occurrence of artificial spectroscopic gap at Dirac point. This problem was noticed and addressed e.g. in Ref. S1 (see Figs. S5 and S6 there).

In Fig. S4 we compare the Fermi surface maps of BiTeI recorded using 22 eV photon energy at the synchrotron. The left map is recorded using the scanning of electron beam within the lens through the entrance slit by hemispherical analyzer.

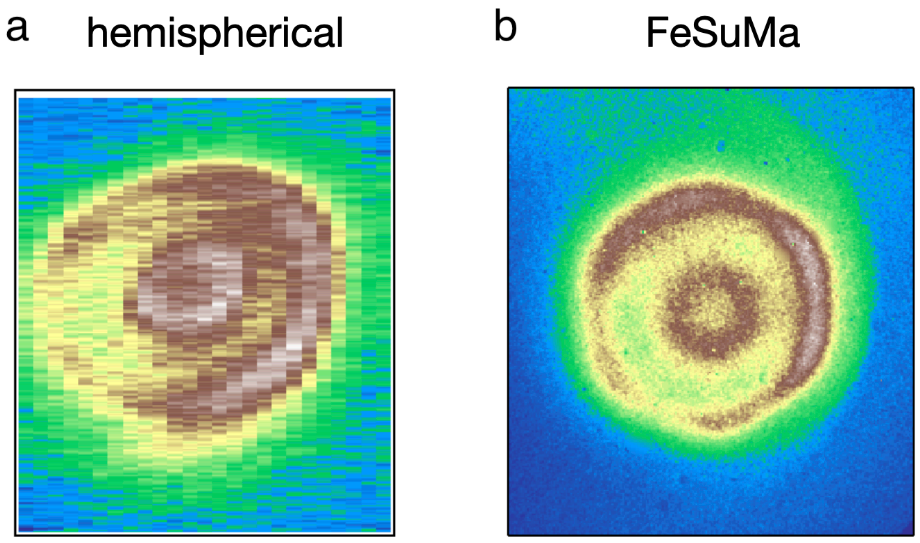

Fig. S4. **Comparison of the Fermi surface maps.** Fermi surface maps of BiTeI recorded using hemispherical and FeSuMa analyzers with 22 eV photons. Intensity is integrated within 20 meV energy window.

a) Conventional Fermi surface mapping within ~15 minutes.
b) FeSuMa Fermi surface map taken within 30 seconds.

Supplementary References

[S1] M. Krivenkov et al. „Origin of the band gap in Bi-intercalated graphene on Ir(111)“, 2D Mater. **8**, 035007 (2021).
