# Supplementary material for: Fermi surface tomography
Source: Nat Commun. 2022 Jul 15;13:4132. doi: 10.1038/s41467-022-31841-z (PMC9287296; doi:10.1038/s41467-022-31841-z)
Supplement: Supplementary file 3 — Description of Additional Supplementary Files [file 41467_2022_31841_MOESM3_ESM.pdf]

Description of Additional Supplementary Files

File Name: Supplementary Movie 1
Description: Fermi surface contour of topological surface states and central bulk sheet in Bi2Te3 in real time.
